# The effect of competition on heart rate during kart driving: A field study

**DOI:** 10.1186/1756-0500-4-342

**Published:** 2011-09-09

**Authors:** Kenta Matsumura, Takehiro Yamakoshi, Yasuhiro Yamakoshi, Peter Rolfe

**Affiliations:** 1 Department of Adult Mental Health, National Institute of Mental Health, National Center of Neurology and Psychiatry, Tokyo, Japan; 2 School of Mechanical Engineering, College of Science and Engineering, Kanazawa University, Kanazawa, Japan; 3 Graduate School of Natural Science & Technology, Kanazawa University, Kanazawa, Japan; 4 Graduate School of Science and Technology, Chiba University, Chiba, Japan; 5 Oxford BioHorizons Ltd, Oxford, UK; 6 Department of Automatic Measurement and Control, Harbin Institute of Technology, Harbin, China

## Abstract

**Background:**

Both the act of competing, which can create a kind of mental stress, and participation in motor sports, which induces physical stress from intense g-forces, are known to increase heart rate dramatically. However, little is known about the specific effect of competition on heart rate during motor sports, particularly during four-wheel car driving. The goal of this preliminary study, therefore, was to investigate whether competition increases heart rate under such situations.

**Findings:**

The participants drove an entry-level formula kart during two competitive races and during solo driving against the clock while heart rate and g-forces were measured. Analyses showed that heart rate values during the races (168.8 beats/min) were significantly higher than those during solo driving (140.9 beats/min) and rest (75.1 beats/min).

**Conclusions:**

The results of this preliminary study indicate that competition heightens heart rate during four-wheel car driving. Kart drivers should be concerned about maintaining good health and developing physical strength.

## Introduction

Accumulating evidence shows that heart rate (HR) dramatically increases during motor sports. For example, Yamakoshi et al. [1] showed that mean HR during racing kart driving was elevated to 150 beats/min, and that this level was maintained over almost the entire 35 min driving period. Recent studies have tended to attribute such HR increases to intense physical demands of high-speed driving, as previously proposed by Falkner [2,3]. The rationale behind these suggestions was that blood lactate concentration immediately after motor sports [4-6] and VO_2 _during motor sports [7,8] consistently increased. However, such a view does not necessarily rule out the involvement of emotional stress, particularly that induced by competition as previously proposed by Schwaberger [6], and Taggart and Carruthers [9,10].

In most sports, the process of competing involves both physical and mental efforts. In motor sports, however, it would appear that the aim to win arises primarily through mental rather than physical effort [6,9,10]. Nevertheless, appropriate physical strength to deal with the isometric challenge [3] presented by intense g-forces [1] is of course necessary. It is striking that psychophysiological studies have shown that such mental effort provokes *β *adrenergic sympathetic nerve activation, typically measured as increased heart rate, blood pressure, and cardiac output [11,12], although such *β *adrenergic activation was often accompanied by parasympathetic withdrawal [13]. For example, a laboratory study using a motorized toy racing car game task conducted by Harrison et al. [14] and Veldhuijzen van Zanten et al. [15] showed that heart rate during the task is higher in the competition condition than that in the solo and co-operation conditions. Thus, unless intense g-forces increase HR to a maximum level, competition induced mental stress during motor sports should increase heart rate.

The rationale for our present study arises from this background concerning the possible competitive factors influencing cardiovascular responses in motor sports athletes. To date, there are only two reported motor sports studies that examined the heart rate during both competitive and solo conditions [4,5]. These studies had well-controlled experimental design, and their results were in accordance with our prediction. That is, regardless of engine size, reflecting required physical demands, HR during competition is higher than that during free run and qualifying sessions. However, these findings were obtained only from motorcycles, so the generalizability of these results to four-wheel car driving is still unknown. Plainly, in general terms both are motor sports, but in fact there are many differences between them, such as the operability of the machine, the very different seating, the influences of the intensity and vector of g-force on the body, and the risks of injury in the event of an accident. Therefore, the purpose of this study was to conduct a preliminary examination of the effect of competition on heart rate during four-wheel car driving by comparing HR during competitive and solo driving.

## Methods

### Participants

A total of 3 men participated in this study. All were amateur kart drivers with an SL kart license certified by the Sport & Leisure Organization (SLO) Corporation in Japan. None of the participants had any current or past history of drug or alcohol abuse. Informed consent was obtained from all participants. This study was approved by the Institutional Review Board of Kanazawa University.

### Field Setting and Conditions

The four-wheel machine used in this study was a CRG sports kart, being an entry-level formula car, consisting of a CRG chassis (KALIFIRNIA, CRG Corp.), four cycle engine (KX21, SUBARU Corp.), and rain tyre (DFK2, Dunlop Corp.).

In the race situation, the three participants formed a team called "team yu.sys", which took part in a sports kart race series, held on July 20 and December 6, 2009, at Biwako (Lake Biwa) Sports Land, Japan. This series of races was of the endurance type, the first lasting 2 hours and the second 3 hours. As part of both races, there were 30 min practice sessions running in the morning, and then the real race began in the afternoon. In the first race, all participants drove the kart once for approximately equal periods of time amounting to about 40 min. continuously. In the second race, all teams were required to change drivers in turn, according to their mean weight, to make race conditions similar. Team yu.sys had seven changes imposed upon it; so all participants drove the kart at least twice. The order of drivers was the same as in the first race, and the driving period ranged from 10 to 30 min.

In the solo driving condition, each participant in team yu.sys drove the kart in the morning on the day after the second race, using the same circuit on which the race had been run. The team members drove in turn, attempting to record their best lap time. Each one drove the kart for about 15 min, at least two times, and the order of the drivers was the same as during the second race.

For the baseline condition, each participant in team yu.sys sat quietly on a chair in a lab, for about 10 min in the morning on the day after the solo runs took place.

In the analysis of our results for the additional solo condition, we have also incorporated some data obtained from a previous kart study [1]. Although that study was conducted on another circuit and used a racing kart that was of a superior class to that used in the present study, other aspects such as the solo driving condition, the driving duration per condition (about 40 min), the drivers' amateur status, the kart experience (4.8 ± 4.4 years), and their mean age (33.5 ± 8.2 years, *N *= 11) and their sex (all were men), were similar to those of the present study.

### Measures and Data Reduction

A measurement instrument, the Active Tracer AC-301A (GMS Corp.), was attached to the driver and used to record the ECG and three axes g-forces. This small device had dimensions of 52.0 mm height × 80.0 mm width × 17.0 mm depth, with a weight of 72 grams, and it had a memory large enough to save over 24 hours of data. Beat-by-beat HR and g-forces on the driver sampled every 1 sec were obtained using this device during the first and the second races, the solo drives, and the baseline conditions. Each measure was averaged over a 1 min period, and then further averaged to produce measures for each consecutive period; that is, the first race, the first and the second run in the second race, the first and the second run in the solo driving, and the baseline value. On averaging, some data were removed according to the following criteria: (a) the first 1 min of each consecutive driving period; (b) the last 3 min of each driving period because a 'pit in' sign was given to the driver when time remaining was less than 2 min.; (c) when the safety car was called on to the circuit; and (d) the third run because not all participants drove three times for each condition

A thermohygrometer (TR-72U, T&D Corp.) and a radiation thermometer (IR-TAF, Chino Corp.) were used to measure the environmental conditions. Ambient temperature and humidity and road temperature were measured using these devices every 20 min. during the first and the second race and during the solo drives. Each measure was averaged to produce a value for each period.

### Statistical Analysis

The data for each period were compared statistically by means of one-way repeated-measures analysis of variance (ANOVA). The Greenhouse-Geisser correction was applied to the degree of freedom where appropriate. Tukey's Honestly Significant Difference tests (Tukey *HSD*) for post-hoc comparison were used with a significance level of 5%. The comparisons between additional solo condition and others were conducted using the *t*-test with Bonferroni correction. HR adjusted for g-forces for each period was also analyzed using ANOVA. All analyses were carried out using the SYSTAT 13 for Windows statistical package (version 13.0.5, SYSTAT Inc.).

## Results

The descriptive characteristics of the participants are shown in Table 1. The weather, ambient temperature and humidity, and road condition and temperature are summarized in Table 2.

**Table 1:** Descriptive characteristics of the participants

Participants	Gender (M/F)	Age (yr)	BMI (kg/m^2^)	Smorker (Y/N)	Kart Experience (yr)
A	M	31	18.8	Y	10
B	M	34	21.3	N	1
C	M	35	21.1	N	3

**Table 2:** Summary of environmental conditions

Period	Weather	**Air Temp**. (°C)	Humidity (%)	Road	**Road Temp**. (°C)
1^st ^Race	Cloudy	27.5 ± 0.8	80.1 ± 4.1	Dry	34.4 ± 1.5
2^nd ^Race					
1^st ^run	Fine	12.0 ± 0.5	45.4 ± 2.7	Dry	23.3 ± 0.7
2^nd ^run	Cloudy	11.4 ± 0.3	43.4 ± 3.8	Dry	20.7 ± 2.0
Solo					
1^st ^run	Drizzly	7.9 ± 0.3	72.8 ± 3.7	Semiwet	12.0 ± 1.0
2^nd ^run	Cloudy	8.4 ± 0.4	67.3 ± 3.0	Semiwet /Dry	10.8 ± 0.9
Baseline	---	---	---	---	---

**Figure 1:**
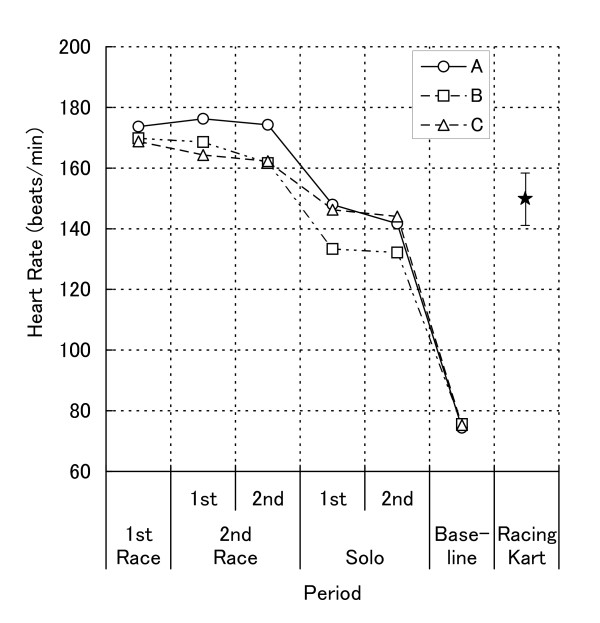
**Mean heart rate as a function of period together with additional solo period (racing kart)**.

Mean and SD values of HR as a function of period are presented in Figure 1. The repeated-measures of one-way ANOVA revealed the main effect of period (*F*_5, 10 _= 190.36, *p *< .001, *ε *= .374, η_p_^2 ^= .99). Subsequent follow-up Tukey *HSD *test showed that regardless of repetition, HR during the first race (170.8 ± 2.6), the first (169.7 ± 6.0) and the second (166.0 ± 7.1) run in the second race period were higher than those during the first (142.5 ± 8.0) and the second (139.3 ± 6.3) run in the solo period, and that HR values during solo periods were higher than those during baseline period (75.1 ± 0.6).

Multiple *t*-tests revealed that, compared to the additional control (152.0 ± 8.2), HR during the first race (*t*_12 _= 3.82, *p *< .05) and the first run in the second race (*t*_12 _= 3.46, *p *< .05) were significantly higher, and baseline (*t*_12 _= 15.73, *p *< .001) was significantly lower.

**Figure 2:**
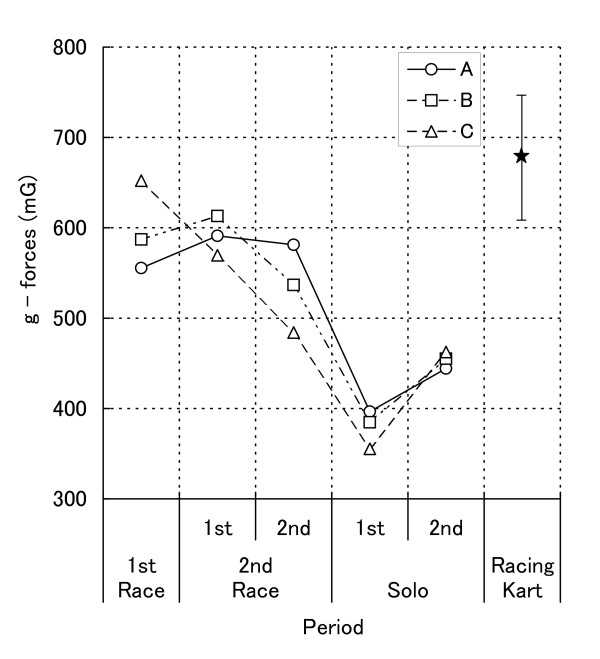
**Mean acceleration as a function of period together with additional solo period (racing kart)**.

Mean and SD values of g-forces as a function of period are presented in Figure 2. The analysis revealed the main effect of period (*F*_4, 8 _= 18.76, *p *< .001, *ε *= .277, η_p_^2 ^= .90). Subsequent post-hoc tests showed that HR during the first race (598.2 ± 49.4), and the first run in the second race (591.3 ± 21.8) period were higher than those during the first run in the solo period (378.8 ± 21.2), but that HR during the second run in the second race (534.0 ± 48.6) was not significantly higher than that during the second run in the solo period (453.9 ± 9.1).

Multiple *t*-tests revealed that, compared to the additional control (681.1 ± 68.7), g-forces during the second run in the second race (*t*_12 _= 3.43, *p *< .05), and the first (*t*_12 _= 7.33, *p *< .001) and the second (*t*_11 _= 10.63, *p *< .001) run in the solo period were significantly lower.

The repeated-measures of one-way ANOVA on HR adjusted for g-forces revealed the main effect of period (*F*_5, 10 _= 23.00, *p *< .001, *ε *= .301, η_p_^2 ^= .92). Subsequent follow-up Tukey *HSD *test showed that HR adjusted for g-forces during the first race, the first and the second run in the second race period were higher than those during the second run in the solo and baseline period, and that HR adjusted for g-forces during the first run in the solo period were higher than that during baseline period.

## Discussion

The aim of this study was to examine the effect of competition on heart rate during four-wheel car driving. To attain this, we conducted field experiments using an entry-level formula car, sports kart, and collected HR data during both races and solo driving against the clock. Statistical analyses of the results showed that HR values during races were higher than those during solo driving as well as at baseline, and that HR values during races were higher than those during solo driving of a higher rank kart machine. These findings suggest that competition heightens HR during four-wheel sports kart driving.

The weather conditions during the solo driving studies were rather poor and so the g-forces recorded were found to be somewhat lower than those found during the racing periods. Since the physical efforts made by drivers in motor sports mainly consist of presenting isometric responses [3] against the g-forces associated with acceleration, braking, and cornering, the difference between HR during races and solo driving might be caused by the difference in g-forces found here. However, we also found that HR during the first and second parts of the solo driving period were comparable, yet the g-forces during the second part were higher than those during the first part. By the same token, we also found that HR during the second period in the second race was higher than that during the second solo period although g-forces during these periods were comparable.

There is also a further point regarding the g-forces. It can be seen that during the additional solo drive the g-forces were higher than those found during the solo condition and as high as the first race and the first run in the second race in the present study, and yet HR during this additional solo condition was as high as that during the present solo condition and lower than those during the first race and the first run in the second race period. In support of this view, the result of statistical analysis on HR was hardly changed at all even after being adjusted for g-forces. Therefore, although the environmental conditions during the additional solo drive were not exactly the same as those during the present study, it does seem justified to attribute the difference between HR during race and solo conditions to the difference in competitive state.

It is well-known that HR is adjusted by *β *adrenergic sympathetic activity and parasympathetic activity, that is to say, the former as accelerator and the latter as brake [16]. Considering Yamakoshi et al. [1]'s finding that both *β *adrenergic activation and approximately complete parasympathetic withdrawal increased HR during solo kart driving, the remaining way to further heighten HR is to further increase *β *adrenergic activation. Thus, the increase in HR from solo driving to racing was highly likely to be due to further *β *adrenergic activation, which is completely consistent with the previous notion that mental effort provokes *β *adrenergic sympathetic nerve activation [11,12].

A race was deemed by us to constitute a competitive situation, but in this particular study such an operational definition might not be entirely valid. This is because the participants formed a team and took part in races together, so these factors must have given rise to a cooperative aspect. However, it has been reported that cooperative situations did not heighten HR [14,15,17], and so this aspect should not falsely strengthen the data in race conditions.

In this study, we have emphasized the possible role of mental effort, that is, competition, within the motor sports context. However, this does not imply that motor sports require minimum physical effort, as previously proposed by Taggart and Carruthers [9,10]. On the contrary, we venture to emphasise the intense physical activity aspect of motor sports. In fact, all participants in the present study reported muscle ache after the experiments. In further research, to quantify physical activity, we will need to measure not only g-forces but also blood lactate concentration or VO_2_, as in some previous studies [4-8]. In addition, it might be useful to measure the EMG during motor sports since this is relatively easy to carry out.

This study was conducted as a preliminary investigation and as such it has been useful. Nevertheless there are two possible limitations. Firstly, although the collected data appeared to be very stable and coherent, the small sample size makes firm conclusions questionable. The conduct of this type of study is by no means straightforward due to the practical constraints of access to suitable race tracks, vehicles and suitable volunteers. Nevertheless, a larger study population will be sought in future work. Secondly, the present data were obtained from one circuit and because circuit characteristics such as roadway and speedway can affect HR [8], data should be obtained from other circuits.

This study has revealed that, in amateur drivers, the mean HR during races approached almost 170 beats/min, which corresponds to over 90% of the maximal HR of the participants. If this were due purely to mental stress, HR would have subsequently been expected to decrease in accordance with the repetition and prolonged continuance phenomena [18,19], but this did not happen in kart driving. These findings indicate that kart driving, particularly under race conditions, is actually a very demanding sport in contrast to its appearance, as previous studies have already mentioned [1,4,5]. Thus, amateur kart drivers should be concerned about maintaining good health, developing physical strength, and only participate in races when fit. This is because maximal VO_2_, one index of physical strength, has been reported to be inversely related to HR during car races [6]. In fact, professional racing drivers have maximal exercise ability comparable to athletes involved in sports such as football, basketball, and baseball [8].

## List of abbreviations

**HR**: heart rate.

## Competing interests

The authors declare that they have no competing interests.

## Authors' contributions

KM conceived and designed the study, performed the experiment and the analysis and drafted the manuscript. TY helped to conceive and design the study, performed the experiment and the analysis, and helped to draft the manuscript. YY performed the experiment and the analysis. RP revised the manuscript critically. All authors read and approved the final manuscript.

## References

[B1] Yamakoshi T Matsumura K Yamakoshi Y Hirose H Rolfe P Physiological measurements and analyses in motor sports: a preliminary study in racing kart athletes Eur J Sport Sci 2010 10 397 406 10.1080/17461391003699112

[B2] Falkner F The stress of racing driving Lancet 1971 1 2700 650 4101257 10.1016/s0140-6736(71)91583-2

[B3] Falkner F Isometric exercise and racing driving Lancet 1972 2 1368 1369 4118234 10.1016/s0140-6736(72)92813-9

[B4] D'Artibale E Tessitore A Capranica L Heart rate and blood lactate concentration of male road-race motorcyclists J Sports Sci 2008 26 683 689 10.1080/02640410701790779 18409099

[B5] D'Artibale E Tessitore A Tiberi M Capranica L Heart rate and blood lactate during official female motorcycling competitions Int J Sports Med 2007 28 662 666 10.1055/s-2007-964889 17455118

[B6] Schwaberger G Heart rate, metabolic and hormonal responses to maximal psycho-emotional and physical stress in motor car racing drivers Int Arch Occup Environ Health 1987 59 579 604 10.1007/BF00377921 3316041

[B7] Gobbi AW Francisco RA Tuy B Kvitne RS Physiological characteristics of top level off-road motorcyclists Br J Sports Med 2005 39 927 931 10.1136/bjsm.2005.018291 16306501 PMC1725105

[B8] Jacobs PL Olvey SE Johnson BM Cohn K Physiological responses to high-speed, open-wheel racecar driving Med Sci Sports Exerc 2002 34 2085 2090 10.1097/00005768-200212000-00033 12471320

[B9] Taggart P Carruthers M Endogenous hyperlipidaemia induced by emotional stress of racing driving Lancet 1971 1 363 366 4100210 10.1016/s0140-6736(71)92207-0

[B10] Taggart P Carruthers M Hyperlipidaemia induced by the stress of racing driving Lancet 1971 1 7704 854 4102539 10.1016/s0140-6736(71)91515-7

[B11] Obrist P Cardiovascular psychophysiology 1981 New York: Plenum Press

[B12] Sherwood A Dolan CA Light KC Hemodynamics of blood pressure responses during active and passive coping Psychophysiology 1990 27 656 668 10.1111/j.1469-8986.1990.tb03189.x 2100351

[B13] Matsumura K Yamakoshi T Rolfe P Love styles and cardiovascular responder types Int J Psychol Stud in press

[B14] Harrison LK Denning S Easton HL Hall JC Burns VE Ring C Carroll D The effects of competition and competitiveness on cardiovascular activity Psychophysiology 2001 38 601 606 10.1111/1469-8986.3840601 11446573

[B15] Veldhuijzen van Zanten JJ De Boer D Harrison LK Ring C Carroll D Willemsen G De Geus EJ Competitiveness and hemodynamic reactions to competition Psychophysiology 2002 39 759 766 10.1111/1469-8986.3960759 12462504

[B16] Berntson GG Cacioppo JT Quigley KS Cardiac Psychophysiology and Autonomic Space in Humans - Empirical-Perspectives and Conceptual Implications Psychol Bull 1993 114 296 322 8416034 10.1037/0033-2909.114.2.296

[B17] Gerin W Pieper C Marchese L Pickering TG The multi-dimensional nature of active coping: differential effects of effort and enhanced control on cardiovascular reactivity Psychosom Med 1992 54 707 719 1454965 10.1097/00006842-199211000-00011

[B18] Kelsey RM Blascovich J Leitten CL Schneider TR Tomaka J Wiens S Cardiovascular reactivity and adaptation to recurrent psychological stress: the moderating effects of evaluative observation Psychophysiology 2000 37 748 756 10.1111/1469-8986.3760748 11117455

[B19] Ring C Burns VE Carroll D Shifting hemodynamics of blood pressure control during prolonged mental stress Psychophysiology 2002 39 585 590 10.1111/1469-8986.3950585 12236324

